# Interglobular Spaces

**Published:** 1878-06

**Authors:** D. C. Hawxhurst


### THE DENTAL REGISTER.

VOL. XXXII.] JUNE, 1878. No. 6.

### INTERGLOBULAR SPACES.

BY D. C. HAWXHURST.

The interglobular spaces of dentine were first described by Czermak. That part of dentine which contains them is designated by Tomes as the granular layer. It consists of globules and interspaces, and might with even greater propriety have been called the globular layer.

If an imperfectly calcified tooth be prepared for microscopic examination by mounting rapidly in inspissated balsam, so as to prevent the spaces from being filled, various spherical and rounded forms are seen. Between these rounded spheres or outlines are dark interspaces which are known as the interglobular spaces. They appear to be similar in shape to the fragments left after cutting crackers from the rolled dough. There is an angular fragment left, concave on all its sides, there may be several concavities on the sides, a series of curves. When these globules meet, a triangular space is formed with concave sides entirely isolated from the other spaces. Or there may be a larger space of great irregularity and bounded by many of these curved lines and angles. To demonstrate these, boil a thin slab of dried dentine in wax. The spaces will be filled and rendered opaque; the globules will be transparent.

There is another method of preparation which has some advantages. Stain a slab of dentine with Beale's carmine fluid. By careful manipulation the uncalcified animal substance which now fills the spaces, will be stained, and will be shown in sharp contrast with the unstained globules.

In making your selection of teeth from which to obtain specimens of interglobular spaces, choose from a rachitic child if possible. The interglobular spaces are extremely abundant in the teeth of such children.

Mr. Salter has made the observation that the interglobular spaces are nearly always to be found in the teeth of weakly organized girls with fair hair and thin, transparent skin. In any case of constitutional weakness of the tegumentary system to which the teeth belong, we may look for deficient calcification of dentine; or, take a tooth exhibiting atrophy of the enamel; you will find abundant interglobular spaces beneath the defective part. Syphilitic stomatitis coming on while the teeth are undergoing development (or indeed any form of stomatitis) is likely to result in interglobular spaces; or, if serious and depressing diseases supervene while dentine is being laid down they are pretty nearly sure to interfere with complete fusion of calcification globules. The specimen once obtained it is to be prepared in the ordinary way until it is thin enough to be put up. Now treat as directed above.

The dentinal tubes may be seen passing through the globules and stretching across the interspaces of the calcified matrix. That these tracts take carmine from the staining fluid may be regarded as evidence that they are uncalcified while the globules resisting the staining fluid in the main, conduct themselves like all perfect dentine.

It seems to be generally true that tissues that are not calcified, but are about to become so, resist the action of acid or alkaline reagents more stoutly than those denser tissues which have been completely consolidated by infiltration of lime salts. Thus lacunæ may be entirely dissolved out of bone or cementum and exhibited free from their attachments. The same is true of the half calcified lining of the Haversian canals which are freed from their connection by maceration in acids. Or, the sheaths of Neumann, which are on the verge of calcification, are readily dissolved out of the harder dentine by hydrochloric acid, and may thus be shown passing through the now slimy intertubular substance like pipe stem imbeded in thin mortar.

Now it is a fact that the interglobular spaces will also resist the action of reagents until the tissues about them are all dissolved away and they are quite isolated. And this fact may be taken as evidence that they too have undergone that transformation peculiar to tissues about to become calcified, although the calcifying material has not been deposited. Like the other examples given, they serve to illustrate to us that remarkable power of resistance to corrosive agents which all tissue exhibits just prior to calcification. It is plain that although they have not yet received a deposit of salts, they have undergone those changes that precede calcification.

Thus the interglobular spaces are to be looked upon as tracts of dentine that have not yet become hardened through a deposit of mineral salts. They consist of soft parts of the matrix that have been cut off from further calcific supply and left uncompleted.

In order to understand these curious spaces, we must consider that the calcification of normal dentine goes forward by the formation of globules of hard dentine in that part of the pulp which is at the time undergoing transformation into matrix. These calcification globules first form as points which enlarge evenly and approach each other, finally fusing and blending so completely that all traces of their outlines are obliterated and only perfect dentine remains. We might call these first globules points of ossification. The process differs from that which has been described in bone formation in many ways, and yet there is this in common: calcific matter in each case begins to be laid down in points and spreads from these, the points becoming granules, the granules becoming globules.

In dentine formation the globules are very minute indeed at first, and increase by the laying down of salts in the animal matrix at their surface, thus enlarging them until they touch and blend. As additions continue, the angular spaces are filled, and thus in the perfect tooth no interglobular spaces are left.

If the calcification is arrested by morbid processes or defect of nutrition, the spaces between the globules remain soft and unaltered. When normal processes are resumed, a further formation of dentine may occur, leaving this unfinished, which is thus cut off from the normal supply of phosphatic salts and from complete calcification, and thus the tooth contains millions of globules with soft spaces between instead of being formed of solid dentine.

These imperfectly calcified teeth are found in various stages of imperfection. In some there are but the faintest indications of incomplete calcification, in others there is a moderate degree of porosity in parts of the tooth. In others still a larger part of the dentine seems to be made up of granules and spaces massed together.

I have already called attention to the fact that the dentinal tubuli pass through the interglobular spaces. They may be seen to pass from the globules to the matrix of the interglobular spaces, and make their way into another globule on the other side. Thus they preserve their continuity from pulp to enamel as if there were no uncalcified tracts in the dentinal matrix.

There is no break in the regularity and perfect formation of the soft cartilage of the dentine, but only a failure of the globules of calcification to increase normally up to the point of perfect fusion with each other, so as to obliterate the uncalcified tracts between them. Of course the structural element of these uncalcified spaces are the same before calcification as afterwards. Had normal processes gone forward to completion, they could only have resulted in hardening the previously soft dentine of the interglobular space. Thus calcification produces no structural change. It does, however, produce a very great change in density. This may be seen when a tooth containing interglobular spaces is extracted and dried; the soft contents shrink until the spaces seem empty and take up foreign matters in which they are soaked or boiled.

Mr. Tomes has remarked that the soft contents of these spaces appear sometimes to undergo calcification at a period subsequent to their formation. In this case a record of the original defect is preserved in the tooth. Faint markings remain by which the eye may dimly trace the contour of the globules, and their interspaces now obliterated by the further progress of calcification occurring at some later epoch in life. Teeth which present these characters are said to resist decay even more persistently than those of average normal tissue. It is probable that their structure is more dense. But on the other hand decay finds little to oppose its progress in dentine containing the uncalcified interglobular spaces. The corrosive agents at work take rapid possession of the dentinal areas thus affected. When caries attacks such dentine its ravages are greatly to be feared, for the porous character of the tissue renders it peculiarly easy for the acid agents of decay to make their way into the tissue and withdraw its already deficient lime salts.—*Transaction Michigan Dental Society.*

